# These are not the neurons you are looking for

**DOI:** 10.7554/eLife.39244

**Published:** 2018-07-27

**Authors:** Victor Benichoux, Daniel J Tollin

**Affiliations:** 1 Unit of Genetics and Physiology of Hearing, Department of NeuroscienceInstitut PasteurParisFrance; 2 Department of Physiology and BiophysicsUniversity of Colorado School of Medicine, Anschutz Medical CampusAuroraUnited States

**Keywords:** *Meriones unguiculatus*, patch clamp, electron microscopy, superior olivary complex, interaural level difference, principal neurons, Other

## Abstract

Studies that looked into how the auditory brainstem processes the difference in the intensity of a sound as it reaches each ear may have wrongly assumed which neurons were being recorded.

**Related research article** Franken TP, Joris PX, Smith PH. 2018. Principal cells of the brainstem's interaural sound level detector are temporal differentiators rather than integrators. *eLife* **7**:e33854. doi: 10.7554/eLife.33854

We may not realize it, but a noise to our left will arrive at our left ear a few hundred microseconds before it will reach our right ear. It will also be a few decibels louder on that side. Though we only hear a single coherent sound, our brain exploits these subtle differences in timing and volume (or level) to pinpoint where the noise came from.

These two cues are respectively termed the interaural time difference and the interaural level difference (Figure 1A and B). According to the duplex theory of sound localization (Rayleigh, 1907), each type of cue is used to find the origin of a different kind of sound: low-frequency sounds are located based on interaural time differences, and high-frequency sounds based on level differences. For over a century, this has served as the main framework to examine how we detect where a noise comes from.

**Figure 1.**
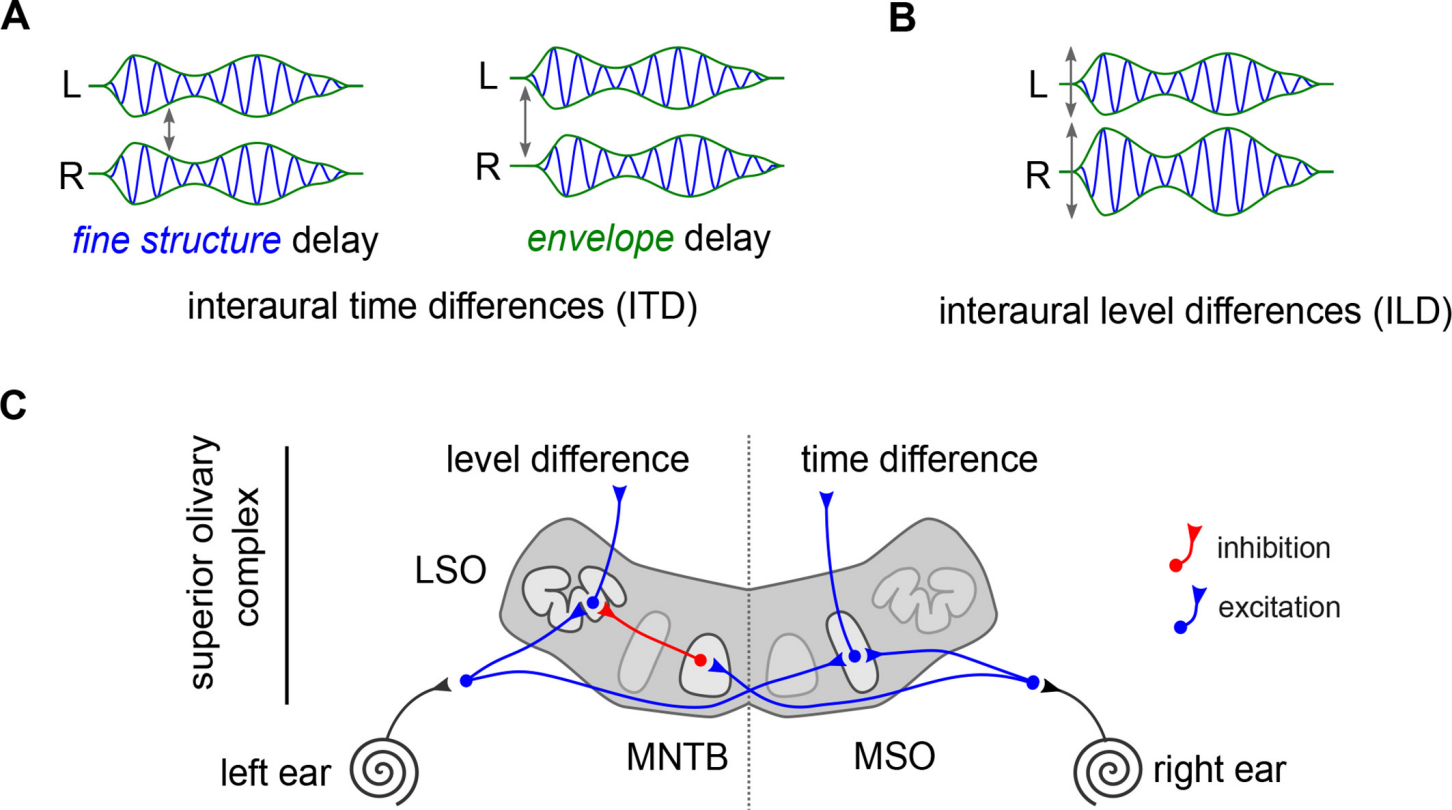
The anatomical implementation of the duplex theory. (**A**) Interaural time differences represent the difference between when a sound reaches the left ear (L) and the right ear (R). They can occur between the fine structure of the sound (blue), or the overall ‘envelope’ of the sound (green). (**B**) Differences in level result from a disparity in amplitudes (height of peaks and troughs) between the two ears (labeled ‘L’ and ‘R’). (**C**) The superior olivary complex receives input from each ear (black lines). The medial superior olive (MSO) receives excitation from both ears (blue lines). The lateral superior olive (LSO) receives excitation from the ear on the same side (blue lines) and inhibition from the ear on the opposite side (red lines) through the medial nucleus of the trapezoid body (MNTB), which is known for its exquisite temporal precision.

In addition, decades of anatomical and physiological studies have explored how this duplex theory could be wired in the brain. These experiments focused on the part of the auditory brainstem where the inputs from each ear first converge: the superior olivary complex (Figure 1C). This area is divided into several structures, which include a medial superior olive (MSO) and a lateral superior olive (LSO) on each side.

Neurons in the medial superior olive (MSO neurons) favor low-frequency sounds. They are also exquisitely sensitive to timing and can react to microsecond differences in the inputs from each ear. MSO neurons accomplish this feat by acting as coincidence detectors. That is, they respond only when excitatory signals from the two ears arrive within a few microseconds of one another. This means that they can detect interaural time differences (Yin and Chan, 1990).

In contrast, the cells in the lateral superior olive (LSO neurons) prefer high-frequency sounds. Extracellular recordings – which measure the electrical signals around the cells – indicated that these neurons respond more slowly, meaning that they could integrate the inhibitory and excitatory signals from the two ears , over a long time window – more than a millisecond (Figure 1C). This would make them able to detect interaural level differences, which could be relayed via changes in the firing rate of the neurons. While there are several types of neuron in the LSO, these characteristics were presumed to belong to the ‘principal neurons’, as these cells form over 80% of the structure.

Now, in eLife, Tom Franken of the Katholieke Universiteit Leuven, Philip Joris (Leuven) and Philip Smith (University of Wisconsin, Madison), report flaws in this assumption (Franken et al., 2018). In an extremely challenging set of experiments, the researchers latched microscopic glass electrodes directly onto individual LSO neurons in the brain of Mongolian gerbils to obtain recordings from within the cells. This was done without knowing whether the cells were principal neurons or neurons of other types. They then characterized how the neurons responded to sound and to interaural level differences. Finally, they identified which of these cells were principal neurons by injecting the neurons with a dye and examining their morphology under an electron microscope.

Together, these data revealed that, contrary to what was observed in the extracellular studies, the principal neurons of the LSO do not exhibit the type of slow response that integrates multiple inputs. Rather, their responses are similar to those shown by MSO neurons (that is, a fast response just to the onset of sound). However, these fast principal cells did still appear to convey information about interaural level differences.

Even before the work by Franken et al., auditory neuroscientists had struggled to directly match the duplex theory onto the superior olivary complex. LSO neurons are inhibited by another area in the brainstem, which encodes the timing of sound with extraordinarily high precision (Figure 1C). In fact, decades ago LSO cells were observed to be sensitive to interaural level differences as well as interaural time differences, which is consistent with this well-timed inhibitory input (Finlayson and Caspary, 1991; Joris and Yin, 1995; Tollin et al., 2005). Nonetheless the view that LSO principal cells are slower and relatively better at integrating signals compared to the MSO has persisted (Remme et al., 2014).

Franken et al. now show that this is not the case, and provide a clue as to why some previous studies may have missed this. Like MSO cells, LSO principal neurons fire off small action potentials, which are difficult to detect through extracellular measures. Thus, prior studies that used extracellular recordings were likely biased towards the other classes of LSO neurons that fire large action potentials; ironically, these cells can integrate signals over time, and also respond to interaural level differences.

To this day, behavioral observations largely support the duplex theory of sound localization (Macpherson and Middlebrooks, 2002). If this framework is not directly reflected in the organization of the superior olivary complex, how is the processing of time and level differences distributed across the LSO and MSO?

Franken et al. suggest that the LSO principal neurons in fact process information in a way that is similar to the MSO: they both act as coincidence detectors. Yet, in contrast to the MSO, the LSO detects the coincidence of excitation from the ear on the same side and inhibition from the opposite side. Rather than conveying information about level differences through changes in their firing rates, the fast LSO cells do so via shifts in the delay of the interaction between excitation and inhibition, which is dependent on the intensity of the sound. Indeed, computational modeling of LSO neurons that detect the coincidence of excitation and inhibition from the two ears was recently shown to effectively create sensitivity to time differences for high-frequency sounds, but also to preserve sensitivity to level differences (Ashida et al., 2017). It is also possible that level differences are processed at the next stage of the brain auditory system; in particular, the neurons in the auditory midbrain integrate inputs over longer time windows, long enough to compute the level-related signals (Brown and Tollin, 2016; Li and Pollak, 2013).

The study by Franken et al. is a good example of how prior expectations can involuntarily mislead scientific endeavor. Indeed, in previous studies, investigators would often classify the neurons that they were ‘blindly’ recording according to predefined sets of expected responses. Franken et al. also cautiously note that, in some prior publications, neurons that exhibited a non-canonical response similar to the one they observed in their own study were typically considered 'pathological' and discarded. Only systematic and rigorous experimental approaches can help detect such anomalies, and rightly cast doubt on well-established scientific dogma.

The new findings now need to be replicated across the species where LSO neurons have been studied to determine whether they will stand the test of time. In the meantime, these results encourage us to pause and rethink how the duplex theory can be implemented in the superior olivary complex.
